# Understanding the Immune Response Changes to Clinical Interventions for Epstein–Barr Virus Infection Prior to Lymphoma Development in Children After Organ Transplants (UNEARTH)

**DOI:** 10.1111/petr.70487

**Published:** 2026-10-09

**Authors:** Vikas R. Dharnidharka, Claire Bocchini, Swati Choudhry, Elizabeth Moulton, Lujain Jaza, Joseph Dumayas, Camila Macedo, Elizabeth Zaragoza, Maryam Mysorewala, Michelle Becker‐Hapak, Mark Foster, Mansi Agarwal, Charles W. Goss, Kenneth B. Schechtman, Kate Nolt, Juan Diego Ramirez, Eileen Rios‐Abdallah, Raja Dandamudi, Michael Green, Kristine M. Wylie, Todd A. Fehniger, Diana Metes, Lara Danziger‐Isakov

**Affiliations:** ^1^ Department of Pediatrics Rutgers Robert Wood Johnson Medical School and Bristol‐Myers‐Squibb Children's Hospital New Brunswick New Jersey USA; ^2^ Texas Children's Hospital and Baylor College of Medicine Houston Texas USA; ^3^ Cook Children's Hospital Fort Worth Texas USA; ^4^ Washington University School of Medicine St. Louis USA; ^5^ University of Pittsburgh School of Medicine Pittsburgh Pennsylvania USA; ^6^ Cincinnati Children's Hospital Cincinnati Ohio USA

## Abstract

**Background:**

Post‐transplant lymphoproliferative disorders (PTLD) remain a major complication of solid organ transplants (SOT), occurring in 1%–10% of patients, but in greater proportions in children. About 50%–80% of cases are Epstein–Barr Virus (EBV) associated, through partially known and still unknown mechanisms. Even in EBV seronaive SOT recipients, transplant of an SOT from an EBV seropositive donor does not always lead to peripheral blood EBV replication. Longitudinal peripheral blood EBV DNA nucleic acid testing (NAT) after SOT has not improved the individual prediction of PTLD occurrence, likely due to variable SOT recipient immune responses.

**Methods:**

This prospective study is designed to assess longitudinal and comprehensive T and NK cell phenotyping and responses to peripheral blood EBV replication via multispectral flow cytometry and other assays. We will also compare local to central EBV NAT values and test whether torquetenovirus NAT can act as a simpler assay to predict EBV clearance. We will collect 1390 blood samples across 5 time points in the first year after 347 enrolled (278 completed) SOTs (kidney, liver, heart, lung or intestine) from three major pediatric SOT centers in the U.S.A.

**Results:**

As of June 30, 2025, our study has enrolled 133 participants, of whom 110 have received an organ transplant (27% Hispanic ethnicity, 16% black race, median age 8 years, across all 4 major SOT types).

**Conclusions:**

By study end, we will know the global immune responses to EBV replication across a spectrum of clinical pediatric SOT situations. We expect to find key immune mechanisms that will predict poor or delayed EBV clearance despite clinical interventions. These findings may lead to new translational immunotherapy approaches to treat EBV DNaemia or prevent PTLD. We expect that our findings will also inform EBV oncogenesis in other immunocompromised or immunocompetent populations.

## Graphical abstract

The T cell and Natural Killer cell immune responses in primary EBV infection in children or during progression to PTLD are not well characterized. By performing detailed prospective immunological phenotyping through the first year after 278 pediatric organ transplants, we will unearth pathways that may inform EBV oncogenesis progression.

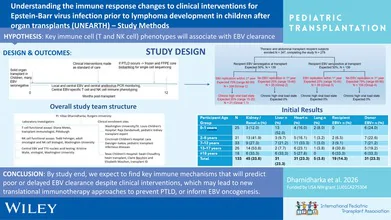

**Abbreviations**
CHVL: chronic high viral load
EBV: epstein–barr virus
NAT: nucleic acid testing
NHLs: non‐hodgkin lymphomas
PBMCs: peripheral blood mononuclear cells
PTLD: post‐transplant lymphoproliferative disorders
PVL: persistent viral load
SOT: solid organ transplants
TEX: terminally exhausted T cells
TPEX: precursor exhausted T cells
TTV: torque teno virus

## 1 Introduction

Epstein–Barr virus (EBV), a ubiquitous herpesvirus, is implicated in approximately 15% of all human cancers, including Burkitt's lymphoma, Hodgkin's disease (HD), nasopharyngeal carcinoma, and gastric carcinoma [1, 2]. Among these, the strongest association is with post‐transplant lymphoproliferative disorders (PTLD), where EBV is linked to 50%–80% of cases [3, 4, 5, 6]. PTLD is a major, life‐threatening complication of solid organ transplantation (SOT), particularly in immunocompromised hosts, and encompasses a spectrum of lymphoid proliferations including non‐Hodgkin lymphomas (NHLs) and Hodgkin lymphomas [3, 7, 8].

Children undergoing SOT are disproportionately affected by PTLD compared to adults [9]. This elevated risk is partly due to the lower baseline incidence of lymphomas in immunocompetent pediatric populations, resulting in dramatically higher standardized incidence ratios—up to 300‐fold for NHLs post‐transplant [10]. A key driver of this risk is primary EBV infection occurring in the context of maximal immunosuppression. Unlike adults, who are typically EBV‐seropositive prior to transplant, children are often seronegative and acquire EBV either through the allograft or community exposure post‐transplant. This primary infection under immunosuppression can lead to uncontrolled viral replication and increased risk of PTLD.

To mitigate this risk, pediatric transplant centers routinely monitor EBV DNA levels in peripheral blood, aiming to preemptively reduce immunosuppression when viral replication is detected [11]. However, this strategy is only partially effective and carries the risk of acute allograft rejection. Emerging therapies such as EBV‐specific T cells are under investigation but remain experimental and limited in availability [12, 13, 14, 15].

A unique phenomenon observed in pediatric SOT recipients is the development of a chronic high viral load (CHVL) state, defined by persistently elevated EBV DNA levels over months [16]. This state is notably absent in adult transplant recipients. Using the Pittsburgh definition— > 16 000 genome copies/mL in ≥ 50% of samples over a 6 month period—CHVL is observed in 8% of pediatric kidney, 18% of liver, and 28% of heart transplant recipients [16, 17, 18, 19, 20]. Importantly, CHVL in thoracic transplant recipients is associated with a 45% progression rate to PTLD, compared to < 10% in abdominal organ recipients.

The mechanisms underlying CHVL and its progression to PTLD remain poorly understood. Preliminary data suggest that CD8+ T cell immunity is distinctly altered in thoracic CHVL patients [21, 22, 23], with evidence of terminally exhausted T cells (TEX), while abdominal CHVL patients may harbor precursor exhausted T cells (TPEX).

T cell exhaustion, characterized by diminished cytokine production and upregulation of inhibitory receptors such as PD‐1, is a hallmark of chronic viral infections and cancer [24]. Key unanswered questions include: how viral loads are maintained, whether CD8+ or CD4+ T cell phenotypes differ between thoracic and abdominal CHVL cases, how immunosuppression influences these immune profiles, and whether predictive biomarkers can be identified. Understanding these dynamics in pediatric SOT recipients with CHVL could illuminate pathways of EBV persistence and PTLD development.

Despite routine EBV DNA monitoring, individual prediction of PTLD remains elusive, likely due to variable immune responses. Our team has explored aspects of T cell exhaustion and viral co‐infections, but a comprehensive evaluation of T and NK cell responses during primary EBV infection and CHVL development has not been performed. This study aims to prospectively characterize immune changes in pediatric SOT recipients during the first year post‐transplant, with the goal of identifying immunologic signatures predictive of PTLD progression. These insights may inform clinical strategies for early intervention and have broader implications for EBV‐associated malignancies in immunocompromised populations.

## 2 Specific Aims

1. Assess the prospective phenotypic and functional profiles of T cell “exhaustion” in pediatric SOT patients during (a) primary EBV infection; and (b) T cell memory generation, resulting in either normal latency (absent EBV loads) or CHVL state.

*SOT recipients' that develop CHVL state display distinct phenotypic memory differentiation and heterogeneous exhausted CD8+ and CD4+ T cell profiles that are regulated by distinct inflammatory circuits. In contrast, patients with absent viral loads will display* a *normal distribution of naïve and anti‐viral functional memory CD8+ with adequate CD4+ T cell help*. We will accomplish this aim by performing multi‐spectral flow cytometry to characterize T cell phenotype and differentiation status, and Meso Scale Discovery platform to assess distinct viral control‐relevant cytokines/chemokines during the phases of initial replication, expansion, progression, CHVL or recovery states.

2 Prospectively define the number, phenotype, and functional status of NK cells in abdominal and thoracic solid organ transplantation patients, and correlate with EBV infection outcomes.

*NK cell activation will coincide with primary infection, will positively correlate with clearance, and negatively correlate with persistent EBV replication. NK cell dysfunction will develop in patients with CHVL, who are at highest risk of PTLD. We will leverage our established NK cell‐focused multi‐spectral flow cytometry panel to analyze a prospective cohort of different EBV response groups after SOT, for questions about the activation status, NK receptor repertoire, and functional capacity. We expect our data to reveal key elements of the NK cell response to primary EBV, and translational approaches to reverse NK cell dysfunction. Further, we will perform functional NK cell experiments from subsets of patient samples to validate that NK cells have altered functionality. T cell and cytokine results from Aim 1 will be correlated with NK cell results obtained in this Aim*.

3 Determine the association of peripheral blood torquetenovirus (TTV) DNA loads with (a) detection of primary EBV infection and (b) development of CHVL state.

*TTV (the dominant human anellovirus) is a published marker of over‐immunosuppression and TTV loads will reduce with clinical reductions in immunosuppression and associate to EBV clearance, while persistent high TTV loads will associate to CHVL*. We will accomplish this aim using longitudinal whole blood NAT PCR assays for both viruses at common time points, performed centrally to minimize lab variability.

## 3 Methods

### 3.1 Participants

Participants will be recruited from three high‐volume pediatric transplant centers, each performing kidney, liver, heart, and lung transplants: Baylor College of Medicine/Texas Children's Hospital, University of Cincinnati/Cincinnati Children's Hospital, and Washington University School of Medicine/St. Louis Children's Hospital. We have obtained Institutional Review Board approval for these studies at all the sites.

Eligible subjects will be identified from the patient populations of the principal investigator (PI) and sub‐investigators (Sub‐I), as well as through referrals from transplant staff at both pediatric and adult SOT programs.

Recruitment will occur prior to transplantation, ideally at the time of listing, to facilitate baseline sample collection at the time of transplant, according to good clinical practice and the Declaration of Helsinki.

### 3.2 Inclusion Criteria

- Recipients of a first or repeat solid organ transplant (single or multi‐organ) at one of the three participating sites.
- Age 0–25 years at the time of transplant.
- Informed consent obtained prior to transplantation.

### 3.3 Exclusion Criteria

- History of hematopoietic stem cell transplant, either prior to or concurrent with the organ transplant.
- Inability to return to the transplant center for scheduled study visits at months 0, 2, 4, 6, and 12.
- History of malignancy, except for hepatoblastoma, which is permitted.

### 3.4 Schedule of Measurements

Each recruited subject will be enrolled prior to transplant, preferably at time of listing, such that we can collect time 0 samples at transplant. For all study subjects, we will collect blood and peripheral blood mononuclear cells (PBMCs) samples at 5 key points post‐transplant—prior to or within 7 days post‐transplant and at Months 2, 4, 6 and 12 post‐transplant. (Figure 1).

**FIGURE 1:**
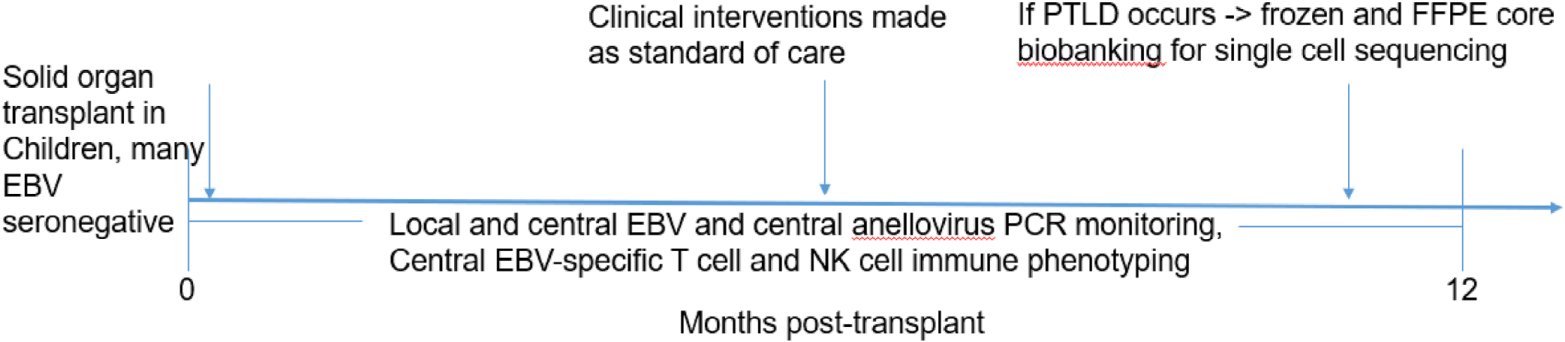
Timeline of study procedures: Study samples will be collected at 5 different time points—at transplant, at 2, 4, 6, and 12 months post‐transplant.

### 3.5 Study Groups

Our study groups (Figure 2) include: (1) EBV seronegative SOT recipients with at least one quantifiable EBV NAT load post‐SOT (Group 1); [2] EBV seronegative SOT recipients that never develop an EBV NAT load in the first year post‐SOT (Group 2); [3] EBV seropositive SOT recipients with at least one quantifiable EBV NAT load post‐SOT (Group 3); [4] EBV seropositive SOT recipients that never develop an EBV NAT load in the first year post‐SOT (Group 4; 5) EBV seronegative SOT recipients that do develop EBV infection AND develop PVL or CHVL (Group 1.1). Note that PVL or CHVL has never been seen in recipients who are EBV seropositive at transplant. The projected sample sizes were chosen based on our centers' collective past 5 years of EBV serology data, the lead site data for EBV replication, and literature estimates of CHVL proportions [16, 17, 18, 19, 20].

**FIGURE 2:**
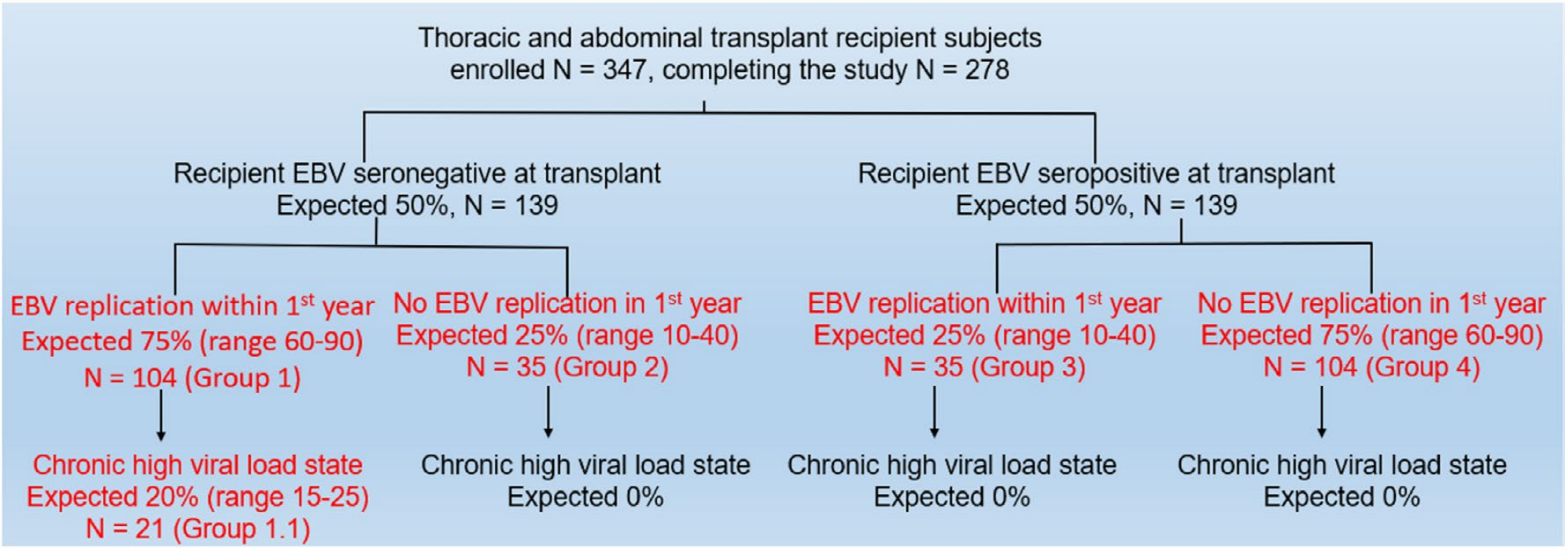
Study groups with projected sample sizes, assuming 50% of recipients will be EBV seronegative at time of transplant, of whom 75% are expected to show EBV replication and a subgroup of 20% will have a chronic high viral load state.

## 4 T cell function assays (performed at the Metes Laboratory at the University of Pittsburgh)

We will use deep phenotyping with Cytek Aurora spectral flow cytometry on banked PBMCs to assess prospectively (from pre‐SOT to 1 year post SOT) the frequency, phenotype and differentiation status of peripheral blood CD8+ and CD4 + T cell compartments, including EBV‐specific cells, in thoracic and abdominal SOT recipients that are EBV negative at transplant (T0), during EBV primary infection (2 m, 4 m), and during memory establishment (6 m, 12 m). We will test 1250 samples from 270 subjects (5 samples in each subject, plus some healthy controls). EBV‐specific CD8+ and CD4+ T cells will be identified with dextramers encoding for the most frequently expressed HLA allele (~75% from general population in North America) and incorporating representative lytic‐ and latent‐derived peptide epitopes [25, 26]. We will stain for N and T_MEM_ subset distribution and for signatures of exhaustion in each patient and cohort [27]. We will identify terminally exhausted (T_EX_) cells as PD‐1+ CD127‐T‐bet‐Eomes+TCF1‐Tox+ and precursor of exhausted (T_PEX_) cells as PD‐1+ CD127+ T‐bet+Eomes+/‐TCF1+ Tox‐ [28, 29, 30]. Our own preliminary data provide evidence of important differences in the CD8+ T cell subsets, with unique or missing cell clusters among patient groups on t‐SNE maps; significant CD8+ T cell restricted cell cluster diversity paralleled by increased T_EM_ activation with IL‐21R+ expression and TEX (PD‐1 + CD127‐ Eomes+) CD8 + T cells detected in thoracic CHVL carriers as compared to those of abdominal CHVL SOT where we detected IL‐21R‐ T_EM_ and T_PEX_ (PD‐1+ CD127+ T‐bet+).

We will also monitor expression of other inhibitory receptors such as LAG‐3, TIM‐3, and CTLA‐4 [84]; and include Ki‐67 and CD31 to determine ex vivo homeostatic proliferative profiles as well as to identify recent thymic emigrants. This is important since our patients in both cohorts receive T cell depleting induction therapy (leading to protracted increased CD4+ T_EM_ levels) or others in the thoracic cohort undergo partial or total thymectomy (responsible for decrease in T_N_ cells with accumulation of oligoclonal PD‐1 + CD8 T_MEM_ cells) [31, 32]. We will compare results from the Study groups cited previously. In addition, markers such as CD38, CD28, and CD40L and ICOS will be included to further determine activation, migratory, and co‐stimulatory potential of CD4+ and CD8+ T cells (Figure 3). More importantly, expression of CD25 (IL‐2Ra), CD122 (IL‐2Rβ), CD127 (IL‐7Rα), and IL‐21R will be also monitored as these common chain cytokine receptors are non‐redundant and critically important for homeostasis and survival of memory CD8+ and CD4+ T cells [33, 34].

**FIGURE 3:**
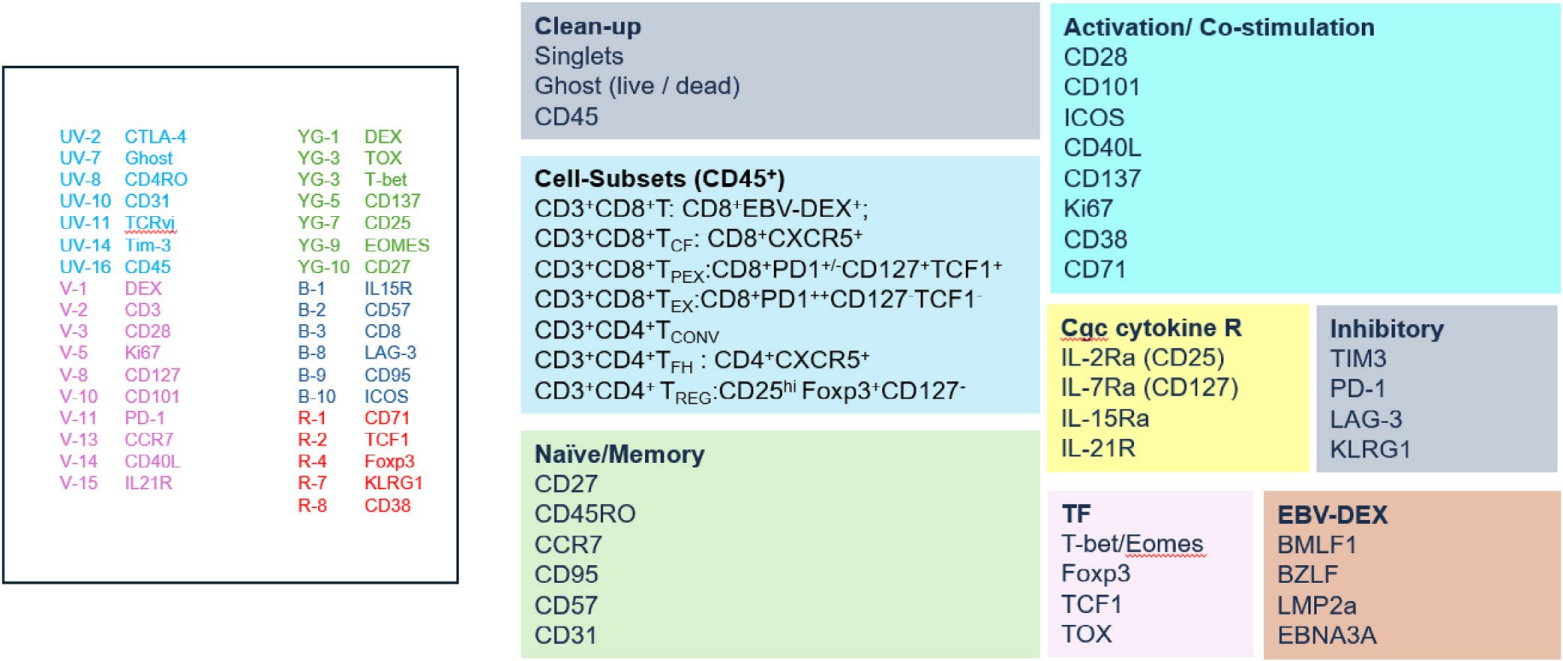
Multispectral flow cytometry panel for T cell phenotyping.

Multiple unsupervised dimensional reduction and integrated analytical pipelines of analysis and clustering (e.g., viSNE, SPADE, FlowSOM, PCA, Kmeans), combined with supervised two‐dimensional analysis (FlowJo), will be implemented with this complex longitudinal data. In selected patients we will set‐up CFSC‐assays with PBMC at longitudinal time points stimulated with commercially available EBV peptide mix specific for (a) lytic Ag (BZLF, BMLF1) to test for EBV reactivation; or for (b) latency Type 2 Ag (LMP1, LMP2, EBNA1) and latency Type 3 Ag (EBNA 3a, EBNA 3b, EBNA 3c), to test for the latency programs present for most EBV‐driven PTLD. In addition, relevant common g chain (γc) cytokine will be added to determine if these receptors are functional during Ag‐specific activation.

We will accurately measure plasma cytokines, chemokines, and soluble factors from pediatric patients at d0, 2, 4, 6, and 12 m time‐points post SOT. We will compare patient groups as described in Aim 1 and identify which soluble factors may distinctly correlate with CD8+ and CD4+ functional T_MEM_ versus T_EX_ generation, allowing or not for chronic high EBV load to occur. Meso Scale Discovery (MSD) is a powerful, high‐performance multiplex technology that measures soluble proteins in plasma and in cell supernatants with high resolution. Specifically, we will focus on cytokines that directly control CD8+ T cell memory differentiation and function, such as IL‐1β, TNF‐*α*, IL‐6, IL‐8, IL‐12, IL‐23 and IL‐10, TGF‐β, Type‐1 IFN. We will also include common *γ* c cytokines (IL‐2, IL‐7, IL15, IL‐21), all measured in plasma or in supernatants of EBV‐stimulated PBMC from each patient group (at time‐points as in Aim1). In addition, we will measure Th‐1, −2, −17 cytokine levels (IFN‐*ɣ*, IL‐4 and IL‐17) as well as relevant chemokines IP‐10, MCP‐1, 4, MIP‐1a, MIP‐1b, MIP‐3a (that help the T cell monocytes/DC cross‐talk) or CXCL13, CCL20, CCL3 CD40L, TNFSF10 (that promote T cell licensing of B cell activation).

A commercially available EBV peptide pool is acquired from Miltenyi Biotech. The PepTivator EBV Consensus pool contains 32 peptides that are MHC Class I restricted and 11 peptides that are MHC Class II restricted. The sequences of these peptides are derived from the following 15 different lytic and latent EBV‐proteins: LMP2a; BRLF1; BMLF1 (EB2); LMP1; EBNA6; EBNA3; EBNA4; BALF2 (DNBI); BMRF1; BZLF1; BNRF1 (MTP); EBNA 1; BLLF1 (gp350); BXLF2; EBNA2. The peptides are restricted for the following HLA‐molecules: HLA−A×02, −A×03, −A×11, −A×24, −A×26, −B×07, −B×08, −B×15, −B×18, −B×27, −B×35, −B×40, −B×4402, −DR.

PepTivator peptide pool has been developed for the efficient in vitro stimulation of EBV antigen‐specific T cells. Here we will perform in vitro stimulation of patients' EBV‐specific T cells by incubating patients' CFSE‐labeled PBMC with PepTivator EBV Consensus peptide pool for 5 days. Subsequently, cells will be harvested, re‐stimulated for 6 h, and stained with different combinations of mAbs that identify surface and intracellular critical T cell markers. Data will be acquired on a Cytec Aurora spectral flow cytometer, and results analyzed by FlowJo software and unsupervised v‐SNE pipeline of analysis using Cytobank.

Functional parameters such as proliferation, effector cytokines/cytotoxic molecules, and up‐regulation of activation/inhibitory markers will be longitudinally monitored on patients' EBV‐specific T cells in response to EBV‐specific stimulation.

This is designed as a T cell study and thus no B cell measurements or B‐cell derived factors have been proposed in the grant.

Data from these experiments will be pre‐processed and normalized with MSD software. We will correlate these data with phenotypic and functional assays as described in Aim1. We expect our results to identify distinct downstream inflammatory circuits with distinct consequences on T cell memory differentiation, function, and/or exhaustion outcomes in thoracic vs. abdominal SOT with normal latency and CHVL carriers. We will adjust for co‐factors that can affect cytokine levels (e.g., organ donor type and source, immunosuppression used).

Using the same multispectral flow cytometry and the MSD pipelines, we will investigate the phenotype of CD8+ and CD4+ T cells (including of EBV‐specific) and plasma cytokine/chemokine levels from CHVL thoracic and abdominal SOT during prophylactic clinical intervention. We will compare results generated from CHVL thoracic and abdominal SOT patients that (a) respond to the intervention or (b) do not respond and may progress to EBV+ PTLD diagnosis. As with Aim 1 and 2, we will also consider as controls (c) the SOT patients who are EBV seronegative but remain EBV NAT negative through the first year; (d) EBV seropositive recipients who remain EBV NAT negative; (e) EBV seropositive recipients who develop quantifiable EBV load but do not progress to PTLD. We will adjust for co‐factors that can affect cytokine levels (e.g., organ donor type and source, immunosuppression used). We will create a Composite score/endpoint based on capturing multiple facets of immune dysfunction/T cell exhaustion as follows: (i) multiparameter phenotypic profiling (PD‐1+, CD127‐T‐bet‐Eomes+TCF1‐Tox+; and co‐expression of additional inhibitory receptors such as LAG‐3, TIM‐3, CTLA‐4); (ii) functional assays (vitro CFSE‐EBV‐specific stimulation of T cells and detection of decreased IFN‐g, TNF‐a, IL‐21, CD107, and elevated IL‐4 and IL‐10 by Flow cytometry); (iii) clinical correlates (clinical asymptomatic chronic high EBV load carrier status, progression to PTLD). This composite measurement may allow us to highlight the multi factorial nature of exhaustion, link immune parameters to patient outcomes and increase the power of statistical analysis.

## 5 NK cell function assays (performed at the Fehniger Laboratory at Washington University School of Medicine)

We will use a panel that is optimized for identifying NK cells using lineage markers and tailored to the questions we address here related to activation and dysfunction (Figure 4). Specifically, we will investigate key aspects of NK cell activation (e.g., CD25, CD69, GzmB, Ki67) within defined subset of NK cells (CD56^bright^, CD56^dim^), as well as the development of dysfunctional NK cells (Eomes, GzmB, TIGIT, LAG3, PD‐1, TIM‐3). Flow cytometry staining, fixation, and sample processing will be performed as described [35, 36]. Spectral flow cytometry data are acquired on Cytek Aurora instrument. Data is collected, stored, then uploaded and analyzed on OMIQ software from Dotmatics using their suite of software tools. We will use dimensionality reduction algorithms to assess NK cells in patients over time, including viSNE [37] and FlowSOM [38, 39].

**FIGURE 4:**
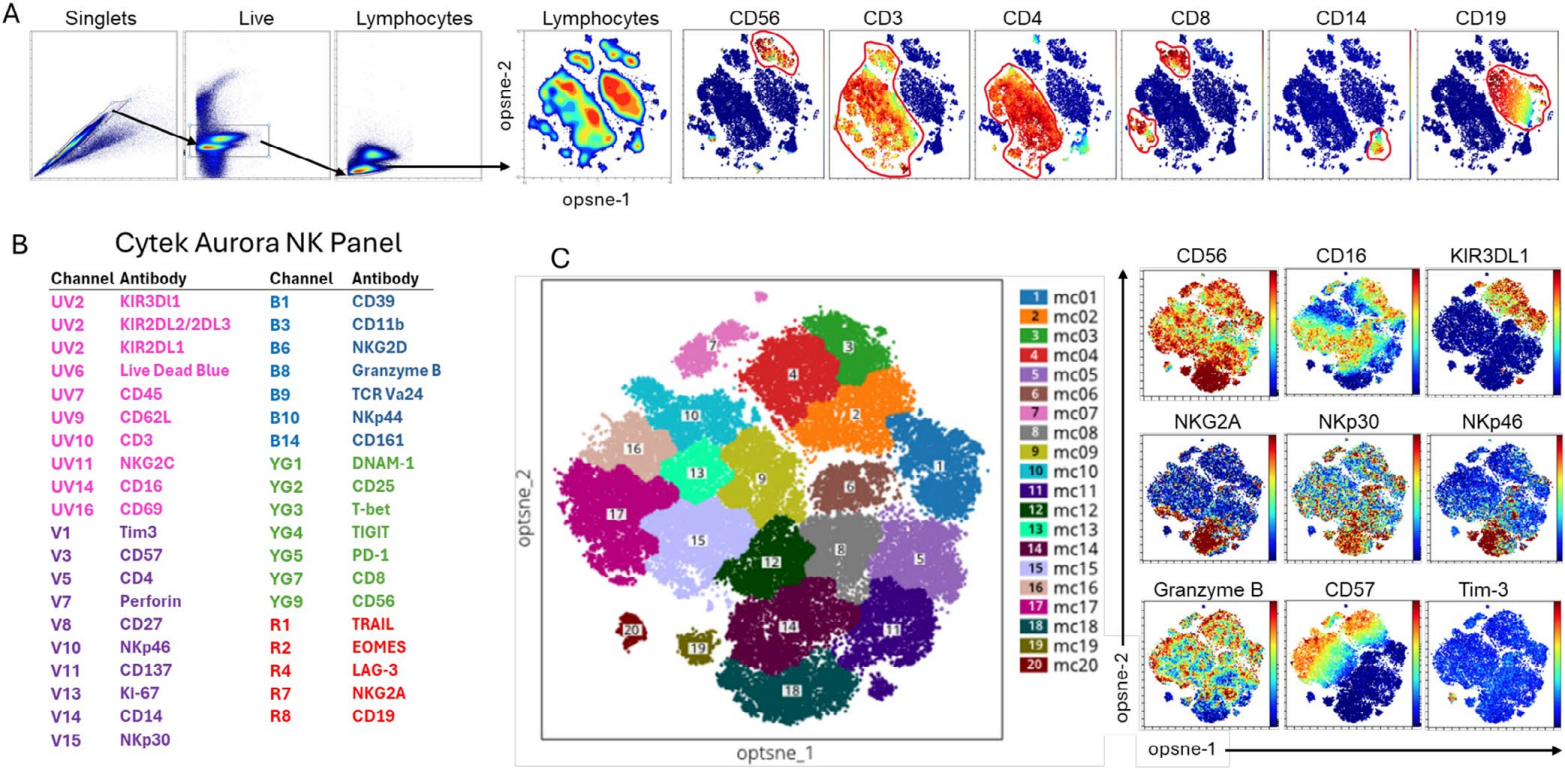
Spectral Flow assessment of thawed PBMC from a normal human donor demonstrating gating and downstream assessments. (A) Concatenated viSNE Contour plots showing canonical peripheral blood components in lymphocyte gate; (B) Spectral Panel marker components using the five laser Cytek Aurora; (C) NK cells (CD45+/CD56+/CD3‐/CD14‐/CD19‐), can be further clustered using FlowSOM into 20 metaclusters to define EBV responsive subpopulations.

## 6 EBV NAT assay methods: (Performed in the Kristine Wylie Lab at Washington University)

Clinical 1 mL whole blood specimens will be collected, frozen immediately, and batched shipped from the sites to the Wylie Lab in blinded fashion. Viral nucleic acid will be extracted by the Maxwell RSC 48 Automated Workstation. Following extraction, specific real‐time PCR reactions are set up on the ABI 7500 real‐time PCR instruments, 5 μL specimen in 50 μL total volume. EBV copy numbers will be determined using a TaqMan PCR assay as previously described [40], which targets a region of the EBNA1 gene, utilizing the EBV international standard. Quantitation will be determined based on comparison to a serial dilution of a 206 bp region of EBNA1 cloned into the pIDTSMART‐AMP vector. The sequences detected by these primers and probe are found within the EBV EBNA‐1 gene: forward primer EBV‐F: 5′ GGT‐AGT‐AAG‐ACC‐TCC‐CTT‐TAC‐AAC‐CT 3′, reverse primer EBV‐R: 5′ TGT‐AAG‐ACG‐ACA‐TTG‐TGG‐AAT‐AGC‐A 3′, probe EBV‐MGB: 5′ 6FAM‐CGA‐GGA‐ACT‐GCC‐C‐MGBNFQ 3′. Limit of detection is < 10 copies per reaction. Levels will be expressed as copies per ml of whole blood. We will use Pittsburgh conversion factors to IU/mL (16 000 genomes = 8200 IU/mL).

## 7 TTV NAT assay methods: (Performed in the Kristine Wylie Lab at Washington University)

To quantify TTV, TaqMan quantitative real‐time PCR will be performed in blinded fashion, using an amplicon that was previously described [41]. The TTV NAT assay we have previously used [42] targets a highly conserved segment of the viral untranslated region. This assay has a 30 copies limit of detection. The quantitation standard consists of a synthesized 144 bp region from the conserved, untranslated TTV genome, which contains the target for PCR, inserted into the pUC57 plasmid. 10‐fold dilutions of the plasmid, starting with 10^8^ copies per ml are used for quantitative standards. TTV levels are expressed as copy number per mL whole blood. For our analyses, we will use published cutoffs > 5.8 × 10^9^ copies/mL that show 90% specificity for infection [43].

## 8 Clinical Data Collection

1. Recipient age, sex, ethnicity, EBV, and CMV serostatus at time of SOT.
2. Type of SOT received, to be stratified in analyses as abdominal or thoracic, initially, then by specific organ type, depending on patient numbers recruited.
3. Donor age, sex, ethnicity, EBV and CMV serostatus at time of organ donation.
4. Induction immunosuppression received or not, and type.
5. Maintenance immunosuppression drugs and dosages/levels at each study time point.
6. Anti‐viral medication type and dosage at each study time point.
7. Any treatments for acute rejection or acute allograft dysfunction.
8. All local post‐transplant CMV and EBV NAT results.
9. Reductions in immunosuppression, use of EBV‐directed T cells, or use of rituximab as interventions for EBV replication.
10. If PTLD occurs within the first year of follow up: location, WHO type, and tumor EBV status if biopsied, all imaging results.
11. Allograft and patient survival till end of the study follow up period.
12. Thymectomy is captured on the transplant form for heart recipients: whether a thymectomy was performed pre‐ or peri‐transplant, the date, and the type (complete/partial).

## 9 Statistical Analysis

### 9.1 Sample Size and Enrollment Estimates

We anticipate that approximately 80% of patients undergoing SOT at the three participating sites will meet eligibility criteria (80% of 217/year ≈174/year). Enrollment will occur over a 3 year period, beginning at Month 6 of the grant and continuing through Month 36 (Figure 5). Each participant will be followed for 12 months, with the final subject completing follow‐up at the end of Year 4.

**FIGURE 5:**
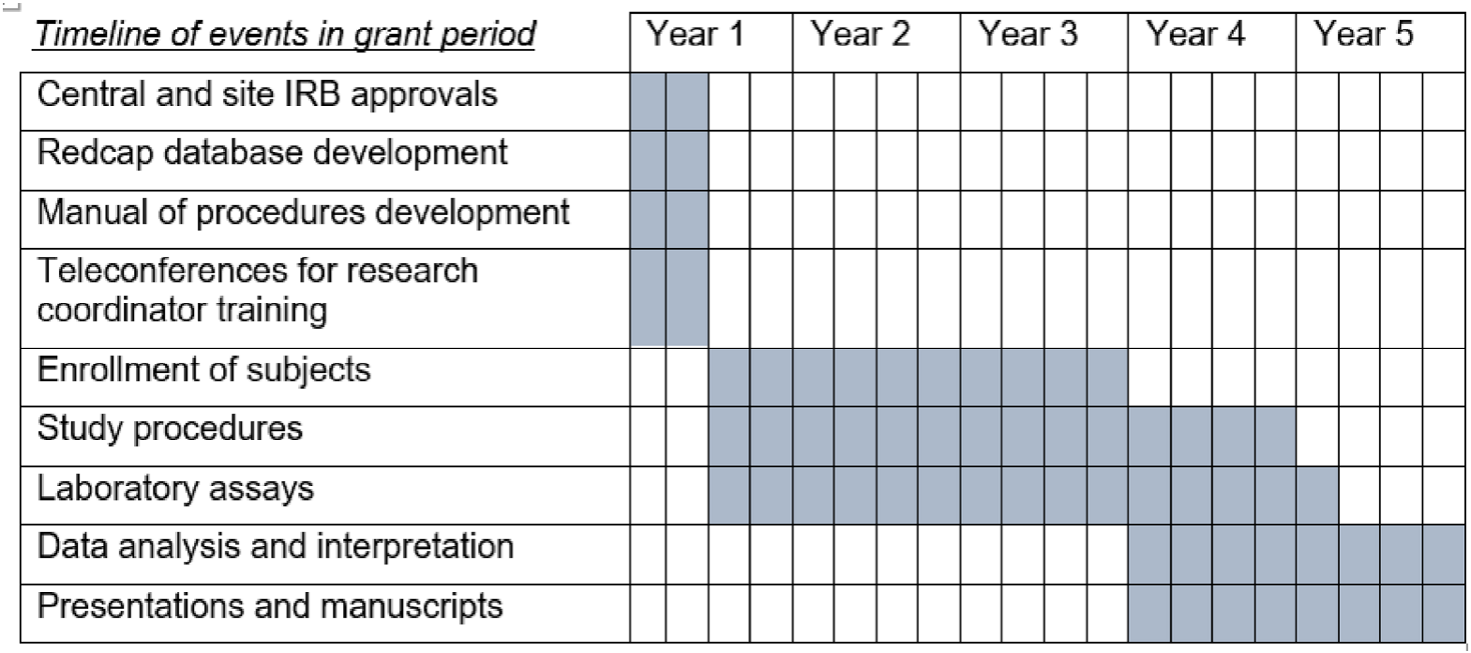
Timeline of study events during the 5‐year grant period.

Based on historical enrollment rates and the low‐risk, observational nature of the study, we expect to enroll 80% of eligible subjects (≈139/year). With lower first‐year mortality and attrition rates in pediatric populations, and the use of incentives and regular contact, we estimate that 80% of enrolled subjects (≈111/year) will complete the full 12‐month follow‐up. Over the 3‐year enrollment period, this yields an expected total of approximately 278 completed participants. The enrollment target was determined from anticipated eligible patient volume, recruitment, retention, and laboratory capacity. For the primary comparison of interest, Group 1 versus Group 4, the anticipated sample of ~104 per group provides 80% power to detect a standardized mean difference of 0.39 or greater (two‐sided α = 0.05). Therefore, the projected sample size for this study should provide sufficient power to evaluate the key hypotheses of interest.

### 9.2 Interim Monitoring and Early Stopping

As this is a non‐interventional observational study, no interim monitoring or early stopping rules will be applied.

### 9.3 Primary Analysis Plan

The primary analyses will focus on EBV replication following initial SOT, which is strongly associated with the development of PTLD. The grouping variable combines recipient EBV serostatus at transplant (positive or negative) and subsequent EBV replication during the 12‐month follow‐up, resulting in four groups.

Outcomes will correspond to the study's three aims:

- Aim 1: T cell phenotypes and function.
- Aim 2: NK cell phenotypes and function
- Aim 3: Peripheral blood torque teno virus (TTV) levels

The primary comparison of interest will be between EBV‐seronegative recipients with replication and EBV‐seropositive recipients without replication. Analyses will evaluate differences in longitudinal immune and virologic measures between these groups. Significant differences (*p* < 0.05) in immune measures (e.g., exhausted T cells, dysfunctional NK cells, elevated TTV) will be interpreted as evidence of impaired immune control in the seronegative + replication group, potentially increasing PTLD risk. Model diagnostics will be performed to assess assumptions of linearity, normality, and homoscedasticity. Transformations (e.g., log) will be applied to skewed variables, and sex will be included as a biological variable where appropriate.

### 9.4 Secondary and Exploratory Analyses

Comparisons involving the remaining EBV groups, transplant type, and cross‐marker associations will be considered secondary or exploratory, depending on patient numbers recruited. CHVL and PTLD will also be examined in exploratory analyses, with interpretation and analysis guided by their observed frequencies.

Longitudinal outcomes will be analyzed using regression models with appropriate distributional assumptions that account for within‐subject correlations over time (e.g., mixed models or generalized estimating equation approaches). Models will include EBV group, time, site, and a time‐by‐group interaction term. We will consider key covariates for inclusion in our multivariable models such as transplant type, immunosuppression regimen, and donor/recipient demographics.

A fifth exposure group—chronic high viral load (CHVL; Group 1.1)—may emerge during follow‐up. Although expected to be small (< 8% of the sample), this group will be included in exploratory analyses. PTLD occurrence (anticipated in 1%–4% of participants) will also be analyzed exploratorily.

CHVL and PTLD will be treated as dichotomous outcomes and analyzed using generalized linear mixed models (GLMMs) with a binomial distribution and logit link. Fixed effects will include site and EBV exposure group; subject will be treated as a random effect. Results will be reported as odds ratios and predicted probabilities. Due to the rarity of these outcomes, covariates will not be adjusted in these models but will be interpreted separately.

We will also explore correlations between NK cell measures and cytokine/T cell populations using Pearson or Spearman correlation at each time point. For longitudinal within‐subject correlations, we will use the rmcorr package in R, which accounts for repeated measures.

### 9.5 Handling of Missing Data

Linear mixed models will be used for primary analyses, which rely on maximum likelihood estimation and provide unbiased estimates under the assumption that data are missing at random. Sensitivity analyses will be conducted as needed to assess the impact of missing data.

## 10 Results

All IRB approvals were obtained and subawards were executed. After development of the final protocol and laboratory manual, we began enrolment. As of June 30, 2025, our study has enrolled 133 participants, of whom 110 had already received an organ transplant. The ethnic and racial stratifications of these participants are shown in Table 1. Hispanic ethnicity is present in 28% and black race in 16%. The enrollment by age and transplanted organ type is shown in Table 2. The median age at enrolment is 8 years, and the participants are divided across all major organ transplant types (kidney 34%, heart and liver 23% each, lung 4%).

**TABLE 1:** Participant enrolment till June 30, 2025 by ethnicity and race.

Racial categories	Ethnic categories									Total
	Not Hispanic or Latino			Hispanic or Latino			Unknown/Not reported ethnicity			
	Female	Male	Unknown/Not reported	Female	Male	Unknown/Not reported	Female	Male	Unknown/Not reported	
American Indian/Alaska native	0	0	0	0	0	0	0	0	0	0
Asian	2	1	0	0	0	0	0	0	0	3
Native Hawaiian or other pacific islander	0	0	0	0	1	0	0	0	0	1
Black or African American	11	10	0	0	0	0	0	0	0	21
White	33	36	0	16	19	0	1	0	0	105
More than one race	1	0	0	0	0	0	0	0	0	1
Unknown or not reported	0	0	0	0	1	0	0	1	0	2
Total	47	47	0	16	21	0	1	1	0	133

**TABLE 2:** Participant enrollment by age group and organ transplant type.

Participant age group	N	Kidney/Renal *n* (%)	Liver *n* (%)	Heart *n* (%)	Lung *n* (%)	Recipient EBV+ *n* (%)	Donor EBV+ *n* (%)
0–1 years	25	3 (12.0)	13 (52.0)	4 (16.0)	2 (8.0)	0	6 (24.0)
2–6 years	31	13 (41.9)	3 (9.7)	5 (16.1)	1 (3.2)	2 (6.5)	7 (22.6)
7–12 years	33	9 (27.3)	7 (21.2)	11 (33.3)	1 (3.0)	3 (9.1)	7 (21.2)
13–17 years	26	14 (53.8)	2 (7.7)	6 (23.1)	1 (3.8)	8 (30.8)	5 (19.2)
≥ 18 years	18	6 (33.3)	6 (33.3)	5 (27.8)	0	6 (33.3)	6 (33.3)
Total	133	45 (33.8)	31 (23.3)	31 (23.3)	5 (3.8)	19 (14.3)	31 (23.3)

*Note:* Median age at enrollment: 8.0 years (IQR: 2.0–15.0). 23 enrolled subjects had not received an organ transplant as of June 30, 2025. Multi‐organ transplants are not listed in the single organ transplant categories above. Three recipients so far received either an intestine only transplant or a liver/intestine/pancreas transplant.

At the onset of the study, we created a laboratory manual and standardized operating procedures. We tested some control samples to optimize cell viability and yield. We have optimized the antibodies for laboratory assays, a necessary step prior to performing the batched laboratory assays.

## 11 Discussion

We have planned for several pitfalls and alternative approaches. We do not anticipate major problems or difficulties with any of the techniques proposed in Aims 1–3, since they are routinely performed in our Labs. With extensive literature on T cell exhaustion during chronic viral infection and cancer, and our own preliminary data, we expect to identify a correlation between T cell phenotype/function and EBV outcomes. We also expect to identify a T cell functional impairment paralleled by distinct inflammatory profiles in thoracic vs. abdominal SOT CHVL. In addition, we expect our proposed studies to reveal important immune signatures relevant for immunologic monitoring in the clinic and to identify novel potential personalized biologic therapies for high risk CHVL SOT patients. Future directions will focus on evaluating whether TEX subsets, if they phenotypically/functionally reverse to TMEM during clinical intervention and CHVL resolve, may display (i) concordant memory transcriptomic and epigenetic circuits, or (ii) preserve exhausted transcripts and open chromatin landscapes influencing future stability of immune responses in these patients [44].

While extensive prior studies have defined a clear role for NK cells in EBV infection response, it is possible that NK cell multi‐spectral cytometry reveals no correlations with EBV outcomes. If this unlikely event occurs, other aspects of the application are examining T cell responses, which would become the primary focus of future research. Classical markers for NK cell dysfunction are not present in transplant patients with EBV replication. We acknowledge that the field of NK cell exhaustion is nascent, and as the field develops we will continue to update our approach to include all potentially relevant markers. In addition, NK cell functional assessments are included, which would be expanded to more patients, allowing for an assessment of NK cell dysfunction. Future directions would include evaluating a subset with NK cell dysfunction and EBV CHVL and controls using our NK cell CITE‐seq assay [45], providing new transcriptomic data to generate hypotheses related to cross‐talk between CHVL and NK cell dysfunction.

## Author Contributions

Vikas R. Dharnidharka: participated in research design, participated in the writing of the paper, participated in the performance of the research, participated in data analysis. Claire Bocchini: Participated in research design, participated in the writing of the paper. Swati Choudhry: Participated in research design, participated in the writing of the paper. Elizabeth Moulton: Participated in research design, participated in the writing of the paper. Lujain Jaza: Participated in the performance of the research, participated in the writing of the paper. Joseph Dumayas: participated in the performance of the research, participated in the writing of the paper. Camila Macedo: participated in the performance of the research, participated in the writing of the paper, contributed new reagents or analytic tools. Elizabeth Zaragoza: participated in the performance of the research, participated in the writing of the paper. Maryam Mysorewala: participated in the performance of the research; participated in the writing of the paper. Michelle Becker‐Hapak: participated in the performance of the research, participated in the writing of the paper, contributed new reagents or analytic tools. Mark Foster: participated in the performance of the research, participated in the writing of the paper. Mansi Agarwal: Participated in research design, participated in the writing of the paper, participated in data analysis. Charles W. Goss: participated in research design, participated in the writing of the paper, participated in data analysis. Kenneth B. Schechtman: participated in research design, participated in the writing of the paper, participated in data analysis. Kate Nolt: participated in research design, participated in the writing of the paper. Juan Diego Ramirez: participated in the performance of the research; participated in the writing of the paper. Eileen Rios‐Abdallah: Participated in the performance of the research, participated in the writing of the paper. Raja Dandamudi: participated in research design, participated in the writing of the paper. Michael Green: participated in research design, participated in the writing of the paper. Kristine M. Wylie: participated in research design, participated in the writing of the paper, contributed new reagents or analytic tools. Todd A. Fehniger: participated in research design, participated in the writing of the paper, contributed new reagents or analytic tools. Diana Metes: participated in research design, participated in the writing of the paper, contributed new reagents or analytic tools. Lara Danziger‐Isakov: participated in research design, participated in the writing of the paper.

## Funding

This research is funded by the NIH/NCI under award number U01CA275304 to Dr. Vikas Dharnidharka.

## Ethics Statement

All studies will be carried out in accordance with the Declaration of Helsinki.

## Conflicts of Interest

Dharnidharka: Research grants: NCI, NIDDK, NIAID. Independent Data Monitoring Committees: Akebia, Takeda. The authors have no conflicts of interest.

## Data Availability

The data that support the findings of this study are available from NIH. Restrictions apply to the availability of these data, which were used under license for this study. Data are available from the author(s) with the permission of NIH.
